# Acute systemic LPS-exposure impairs perivascular CSF distribution in mice

**DOI:** 10.1186/s12974-021-02082-6

**Published:** 2021-01-29

**Authors:** Oscar Manouchehrian, Marta Ramos, Sara Bachiller, Iben Lundgaard, Tomas Deierborg

**Affiliations:** 1 Experimental Neuroinflammation Laboratory, Department of Experimental Medical Science, Lund University, SE-221 84 Lund, Sweden; 2 Department of Experimental Medical Science, Lund University, SE-221 84 Lund, Sweden; 3 Wallenberg Centre for Molecular Medicine, Lund University, SE-223 62 Lund, Sweden

**Keywords:** LPS, CSF, Microglia, Glymphatic System, AQP4, Inflammation

## Abstract

**Background:**

The exchange of cerebrospinal (CSF) and interstitial fluid is believed to be vital for waste clearance in the brain. The sleep-dependent glymphatic system, which is comprised of perivascular flow of CSF and is largely dependent on arterial pulsatility and astrocytic aquaporin-4 (AQP4) expression, facilitates much of this brain clearance. During the last decade, several observations have indicated that impaired glymphatic function goes hand in hand with neurodegenerative diseases. Since pathologies of the brain carry inflammatory components, we wanted to know how acute inflammation, e.g., with lipopolysaccharide (LPS) injections, would affect the glymphatic system. In this study, we aim to measure the effect of LPS on perivascular CSF distribution as a measure of glymphatic function.

**Methods:**

Three hours after injection of LPS (1 mg/kg i.p.), C57bl/6 mice were (1) imaged for two CSF tracers, injected into cisterna magna, (2) transcardially perfused with buffer, or (3) used for physiological readouts. Tracer flow was imaged using a low magnification microscope on fixed brains, as well as using vibratome-cut slices for measuring tracer penetration in the brain. Cytokines, glial, and BBB-permeability markers were measured with ELISAs, Western blots, and immunohistochemistry. Cerebral blood flow was approximated using laser Doppler flowmetry, respiration and heart rate with a surgical monitor, and AQP4-polarization was quantified using confocal microscopy of immunolabeled brain sections.

**Results:**

LPS-injections significantly lowered perivascular CSF tracer flow and penetration into the parenchyma. No differences in AQP4 polarization, cytokines, astroglial and BBB markers, cerebral blood flow, or respiration were detected in LPS-injected mice, although LPS did elevate cortical Iba1^+^ area and heart rate.

**Conclusions:**

This study reports another physiological response after acute exposure to the bacterial endotoxin LPS, namely the statistically significant decrease in perivascular distribution of CSF. These observations may benefit our understanding of the role of systemic inflammation in brain clearance.

## Introduction

Lipopolysaccharides (LPS) are molecules found in the outer membrane of Gram-negative bacteria, and are frequently used in many animal models to mimic inflammatory responses, e.g., in sepsis, depression, and neurodegeneration [1–3]. In 2012, Erickson et al. showed that repeated systemic LPS-exposure lowered bulk flow of CSF, and impaired amyloid-β clearance [4]. The authors thus hypothesized that systemic inflammation could contribute to Alzheimer’s disease [4]. The bulk flow of cerebrospinal fluid (CSF) along periarterial spaces, to be exchanged with interstitial fluid, has since 2013 been known as the glymphatic system [5]. Thereafter, many publications have implicated this sleep-dependent system [6] in the clearance of neurodegenerative protein aggregates [7, 8], but none (to our knowledge) have imaged the direct effect of an inflammatory stimuli on the periarterial CSF-movement. Elucidating how inflammation impairs flow of CSF could give valuable insight into the pathology of common neurodegenerative diseases. Imaging CSF-tracers after Cisterna magna-injections is now a standard method of tracking CSF movement [6, 9, 10]. We used this method to assess the acute effect of a single systemic LPS injection on perivascular CSF dynamics in the mouse. In addition, important facilitators of glymphatic function such as AQP4 polarization and cortical blood flow were measured.

## Materials and methods

### Animals

All procedures, handling, and housing in this study were carried out in accordance with the international guidelines on experimental animal research and were approved by the Malmö-Lund Ethical Committee for Animal Research in Sweden (M250-11, M30-16, Dnr 5.8.18-01107/2018). Male C57BL/6/N mice (age 12 weeks and weight between 23 and 32 g) were acquired from Janvier Labs (France). Mice were housed according to the regulations of Lund University and compliant with the international guidelines on experimental animal research. Mice were group-housed in 12 h dark/light cycle at a stable temperature with access to water and chow, ad libitum.

### Drugs

LPS (1 mg/kg, Sigma, catalog no. L4516, lot 014M4107V or 01M4049V) or its vehicle (ddH_2_O, volume corresponding to that of LPS-injected mice, i.e., 4 μL/g body weight) were injected intraperitoneally, 3 h before (1) the injection of the CSF-tracers into the cisterna magna (LPS *n* = 18, vehicle *n* = 15), (2) transcardial perfusion (LPS *n* = 5, vehicle *n* = 5), or (3) experiments with physiological readouts (LPS *n* = 4, vehicle *n* = 5).

Anesthesia was administered before cisterna magna injections as a mixture of racemic ketamine (Ketaminol®, catalog no. 511519, 100 mg/kg) and xylazine (Rompun®, catalog no. 022545, 10 mg/kg) in 0.9 % saline, referred to as KX, intraperitoneally (i.p.).

Animals for physiological readouts were anesthetized with KX and were redosed with ketamine (50 mg/kg) 1 h after initial dose. Two hours after initial dose, mice were given a third of the initial KX dose, and another ketamine dose (20 mg/kg) after 3 h. Ketamine redosing, as opposed to using more conventional anesthetics, was chosen as not to mix anesthetics.

### Cisterna magna CSF tracer injection

The fluorescent CSF tracers (Alexa-647 conjugated 66 kDa-BSA and FITC-conjugated lysine-fixable 3 kDa-dextran, ThermoFisher) were dissolved together in 0.9% saline at a concentration of 0.5% (w/v).

LPS-treated and vehicle mice were put under general anesthesia and placed in a stereotaxic frame. The cisterna magna was surgically exposed and a 30G needle connected to a 50 μL Hamilton syringe through a polyethylene tube (I.D. 0.28 mm) was inserted in the cisterna magna. Ten microliters of CSF tracer were injected at a rate of 1 μL/min for 10 min with a Harvard apparatus pump. After 30 min circulation time, mice were decapitated and brains were quickly removed and immersed in 4% paraformaldehyde (Histolab), PFA, overnight.

Seven animals in total were removed from glymphatic measurements, due to death during anesthesia, or cerebellar injections, and have not been included in this communication, giving us *n =* 13 for the LPS group and *n =* 14 for the vehicle animals (however, mice with cerebellar injections were still used for later immunohistochemistry).

Brains were imaged using a × 0.5 objective on a SMZ25 Stereo Microscope (Nikon) and tracer distribution was quantified using ImageJ software. Briefly, areas corresponding to cerebellum, cortex, olfactory bulb, and ROI confined to the perivascular space of the middle cerebral artery (MCA area) were outlined with the polygon tool in ImageJ, and fluorescent intensities were measured. Brain coronal slices (200 μm) were sectioned with a vibratome and imaged with a Nikon ECLIPSE Ti2 microscope at × 4 magnification. Tracer penetration was measured in slices using a Fiji [11] macro developed by SciLifeLab Uppsala (anna.klemm@it.uu.se, Supplementary materials) which uses the PerObjectEllipsefit plugin [12], to automatically detect the slice and calculate the threshold and then measures the mean intensity of the slice.

### Laser Doppler and physiological readout

Mice were anesthetized with KX, as described above, and placed on a surgical monitor platform (Harvard apparatus, catalog no. 75-1500, Holliston, USA) fitted with a heating pad and sensors for temperature (kept stable at 37 °C), heart, and respiration rate. Cortical Laser Doppler (Perimed, PeriFlux System 5000, Stockholm, Sweden) flowmetry was measured by surgically exposing the right hemisphere skull and gluing (Loctite, EAN 5010266241173) an 0.5 mm optical filament (Perimed, product no MT B500-0L120), connected to the probe (Ellab, product no PRA22002A275M), on the skull close to the MCA. Once a stable Laser Doppler value could be recorded, LPS (*n* = 4) or vehicle (*n* = 5) mice were injected intraperitoneally, and measurements were recorded every 15 min for 165 min, and then animals were sacrificed. One out of four LPS-treated mice, and two out of the five controls died during anesthesia, and their measurements were thus disregarded from our analyses, giving us *n* = 3 for both groups in the LD/physiological experiments.

### Immunohistochemistry and analysis

Cortical slices (200 μm) used in CSF tracer imaging were also used for immunohistochemical analysis. For IgG extravasation measurements, we used 35 μm cryotome sections from transcardially perfused brain hemispheres. For the immunolabeling with AQP4/Glut1, slices were first rinsed in phosphate-buffered saline (PBS), followed by 3 h block in Normal donkey serum 10% (v/v) in PBS with Tween-20 (Sigma, 0.25% v/v), PBS-T20. Primary antibodies were incubated for 72 h at 4 °C in blocking solution. For immunolabeling, sections were immunoreacted with primary antibodies for AQP4 (rabbit, Merck Millipore, catalog no. AB3594, 1:300) and GLUT1 (mouse, Merck Millipore, catalog no. MABS132, 1:350). After the primary incubation, slices were rinsed 1 h in PBS-T20, followed by blocking solution for 15 min, then 3-h incubation of secondary Alexa Fluor antibodies against rabbit (488 nm, Invitrogen, catalog no. A-11055, 1:500) and mouse (568 nm, Invitrogen, catalog no. A-10037,1:500) in blocking solution. After rinsing in PBS 3 × 5 min, the samples were air-dried and mounted with Diamond Antifade Mountant (ThermoScientific, Sweden). In the 200 μm slices with successful staining, a total of 13 LPS-treated mice and 14 controls were represented and analyzed. The polarization of AQP4 along vessels in tissue was imaged and quantified blindly in the cortex, as described before [10, 13, 14]. Six different blood vessels per animal from comparable regions of the cortex were selected at random and imaged with a Nikon Confocal A1RHD microscope at × 40 magnification. For AQP4 polarization quantification, a cross-sectional line was drawn using the line plot tool in ImageJ and centered on the blood vessel in order to include both AQP4 signal from the vascular endfeet and signal from the parenchyma. The AQP4 polarization was calculated by averaging the peak intensity of the AQP4 signal in the vascular endfeet divided by the average of the parenchymal fluorescence signal, and differences between groups were assessed with an unpaired *t* test.

For the staining against Iba-1, the slices (successfully stained controls *n* = 12, LPS *n* = 14) were first rinsed in PBS, followed by 1 h quench in 1% peroxide dissolved in PBS. After washing with PBS, slices were blocked with glycine (Sigma, 0.5 M) in PBS for 1 h, and then washed with PBS and later PBS Triton X100 (Sigma, 0.5% v/v), PBS-TX, for 30 min. For immunolabeling, the primary antibodies against Iba1 (rabbit, Wako, catalog no. 27981192, 1:20000) was applied for 48 h at 4 °C in PBS-TX. After the primary incubation, slices were rinsed 2 × 30 min in PBS-TX, followed by 2.5-h incubation of secondary antibodies (biotinylated anti-rabbit IgG (Vector), 1 μg/L) in PBS-TX. After rinsing in PBS-TX 2 × 30 min, the samples were treated with ABC elite reagent (Vectastain, catalog no. PK-7100) 1 h, then rinsed in PBS-TX, and thereafter treated with DAB with metal enhancer (Sigma, catalog no. D0426). The reaction was stopped with PBS after 5 min, and slices were mounted on microscope slides, rinsed shortly with Xylene (Sigma) and subsequently mounted with DPX (Sigma). Slices were scanned (Hamamatsu digital slide scanner) and Iba1-positive cells were manually counted in 3 random fields of cortical images in 2 sections (at bregma 0 and bregma − 2 mm) from each animal. Image analysis (Fiji) using the cell-counter plugin were made blinded to assess changes in microglial cell density. In addition, in order to estimate the Iba1^+^ area, we blindly and manually thresholded images to omit the background and measured the Iba1^+^ area fraction in Fiji. For IgG extravasation measurements, the perfused and fixed 35 μm slices were first rinsed in PBS, followed by 2 h block in bovine serum albumin 10% (v/v) in PBS-T20, incubated overnight in 4° with secondary Alexa Fluor antibodies against mouse (488 nm, Invitrogen, catalog no. A-21202, 1:500) in blocking solution. After rinsing in PBS 3 × 5 min, the samples were air-dried and mounted with Diamond Antifade Mountant (ThermoScientific, Sweden), and then imaged with a Nikon ECLIPSE Ti2 microscope at × 20 magnification. IgG intensity was measured in cortical areas corresponding to around bregma 0 mm using Fiji.

### Western blot and cytokine ELISA

LPS-treated (*n* = 5) and control mice (*n* = 5) were transcardially perfused under deep anesthesia with PBS. The brains were removed, snap frozen, and homogenized in RIPA buffer (Sigma-Aldrich, Germany) with proteinase and phosphatase inhibitors (Roche, Switzerland). Later, protein concentration was measured using a BCA kit according to the manufacturer’s protocol (BCA Protein Assay-Kit, ThermoScientific, Sweden), and mixed with 2× LAEMMLI buffer (Bio-Rad, Sweden) and boiled at 95 °C for 5 min. Each well was loaded with 10 μg protein and was separated by SDS-PAGE using pre-cast gels (4–20%, Bio-Rad) in TGS buffer (Bio-Rad, Sweden). For AQP4 blotting, 2 ng of control antigen was diluted in RIPA, mixed with LAEMMLI as the other samples, and loaded together with the protein samples. The proteins were transferred to nitrocellulose membranes (Bio-Rad, Sweden) using the TransBlot Turbo system from Bio-Rad. The membranes were washed 1× with PBS, and subsequently blocked for 1 h with skim milk at 3% (w/v) in PBS 0.1% (w/v) Tween 20 (PBS-T), then washed 3 × 10 min in PBS-T. Then, blots were incubated with primary antibodies in PBS-T overnight in 4 °C, for AQP4 (rabbit, Merck Millipore, AB3594) 1:1000, GFAP (goat, Santa Cruz, catalog no. SC-6170) 1:5000, and Galectin-3 (goat, R&D, catalog no. AF1197) 1:1000.

After washing as abovementioned, the membranes were incubated with peroxidase-conjugated secondaries (Vector, 1:5000) for 2 h RT. After washing 3 × 10 min with PBS-T, the blots were developed using Super Signal West Femto Sensitivity Substrate (ThermoScientific, Sweden) or ECL Clarity (Bio-Rad) according to the manufacturer’s protocol and imaged using the ChemiDoc XRS system from Bio-Rad. For AQP4, blots were imaged using the ChemiDoc XRS+ system (Bio-Rad). After developing the primary blot, actin conjugated with secondary (Sigma, catalog no. A3854), 1:10000 in PBS-T for 20 min, then washed and developed as above. Band intensities were measured in Image Lab software (Bio-Rad) and normalized to actin levels.

MesoScale (MSD) plates were used to evaluate the cytokine levels (proinflammatory panels for IFN-γ, IL-1β, IL-2, IL-4, IL-5, IL-6, IL-8, IL-10, IL-12, and TNF-α) from cortical together with hippocampal homogenates, dissected from both perfused and tracer-injected frozen hemispheres. Analyses were carried out according to protocol, as described before [15].

### Statistical analyses

Tracer experiments were made in two cohorts, 6 months apart, where measurements from the second cohort of controls animals (*n* = 7) were normalized to the first batch of controls (*n* = 6). The fluorescence intensity adjustment index from the controls was then applied to the second batch of LPS mice as well (*n* = 7).

For the arbitrary measurement values of Laser Doppler, values were decimalized (baseline set to 100). For analysis of heart rate, respiration, and temperature, values were instead normalized to the average baselines.

Datasets were assessed for normality, where possible (*N* too low in Western and IgG assays), with D’Agostino and Pearson tests. All sets passed normality testing. Thus, student’s *t* tests were used for comparisons of whole-hemisphere tracer, tracer penetration per animal, AQP4, Iba1, GFAP, galectin-3, IgG, and IL-10 comparisons. For comparison of tracer penetration per animal, we averaged tracer intensity from all slices per animal, decimalized all values, and set the vehicle means to zero—this way treatment differences could be understood as percentages of controls. Differences in tracer area penetration across different brain levels were evaluated with a two-way ANOVA, using multiple comparisons with a Šidák correction. Differences in cortical blood flow, respiration, and temperature were determined with two-way ANOVA. For analysis of heart rate, we used a mixed-effects model (because of missing values for two timepoints in one animal) to assess differences after LPS-exposure compared to baseline.

Statistical analyses were all performed in GraphPad Prism version 8.4.2. *P* values ≤ 0.05 were considered significant; however, differences were also described with 95% confidence intervals to illustrate treatment effects.

## Results

### Cisternae Magna injections

#### LPS injection decreases distribution of CSF tracer in the brain

We assessed the effect of LPS to CSF flow in the brain by injecting two tracers (Alexa-647 conjugated BSA (66kDa) and FITC-conjugated lysine-fixable dextran (3 kDa)) into the cisterna magna, from where they could distribute for 30 min before collection of brains.

**Fig. 1:**
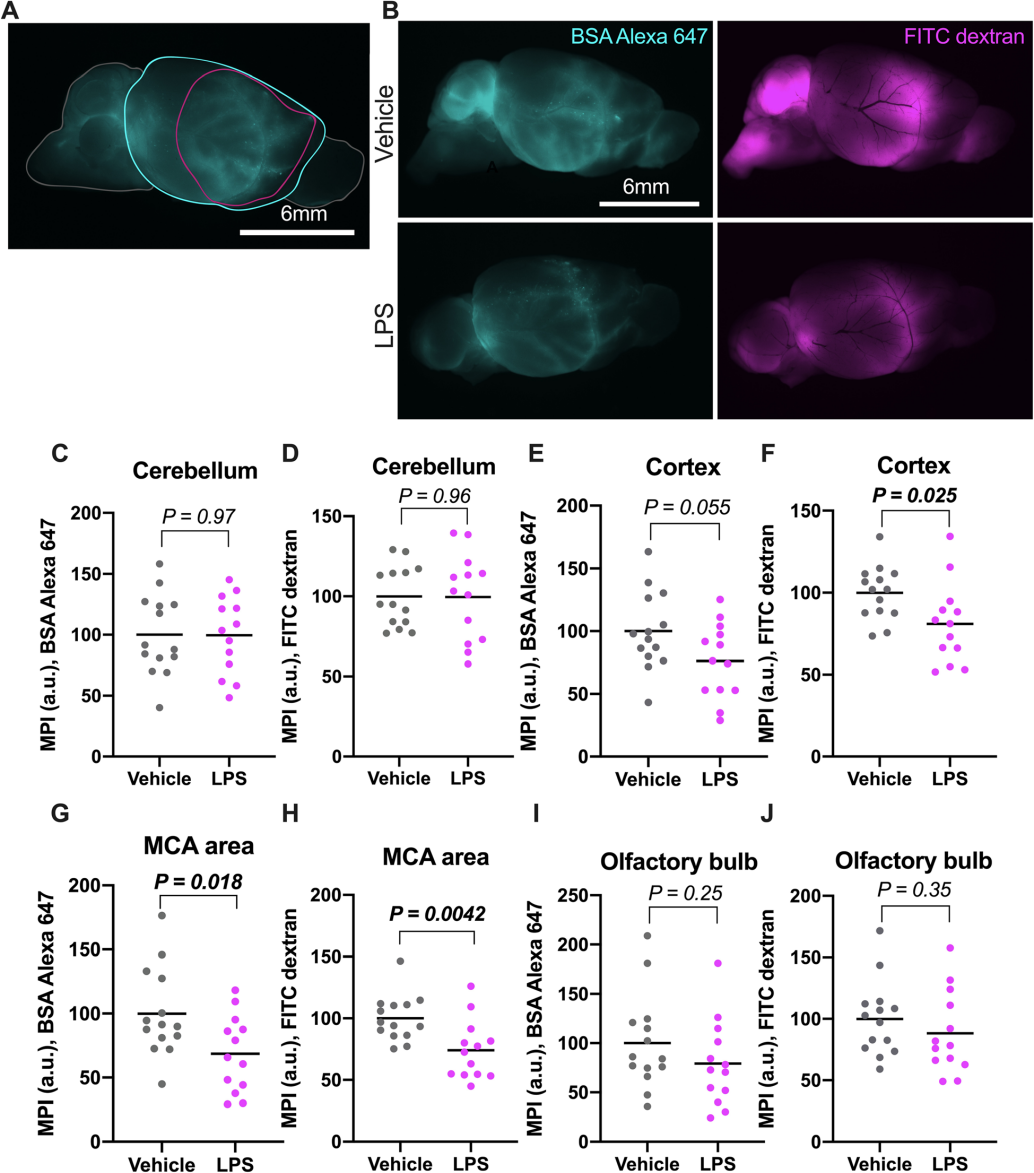
CSF-tracer intensity 3h after systemic LPS-injection (*n* = 14), compared with vehicle (*n* = 13). **a** Color coded areas used for mean fluorescence intensity measurements in ImageJ. Left (grey) cerebellum. Middle (magenta), MCA area (ROI confined to the perivascular space of the MCA). Middle (cyan), cortex. Right (grey), olfactory bulb. Scale bar 6 mm. **b** Representative tracer (BSA Alexa 647 and FITC dextran) images for Vehicle and LPS-groups. **c**–**j** Statistical analyses of mean pixel intensity in arbitrary units, MPI (a.u.). **c** MPI (a.u.) in area corresponding to cerebellum, BSA Alexa 647. Unpaired *t* test, 95% CI of difference = − 26 to 25. **d** MPI (a.u.) in area corresponding to cerebellum, FITC dextran. Unpaired *t* test, 95% CI of difference = − 19 to 18. **e** MPI (a.u.) in whole cortical brain area, BSA Alexa 647. 24% lower in LPS-mice, albeit not significantly so. Unpaired *t* test, 95% CI of difference = − 48 to 0.57. **f** MPI (a.u.) in cortical brain area, FITC dextran is 19% lower in LPS-treated animals. Unpaired *t* test, 95% CI of difference = − 35 to − 2.6. **G:** MPI (a.u.) in MCA area, BSA Alexa 647 was 31% lower on average in LPS-mice. Unpaired *t* test, 95% CI of difference = − 57 to − 5.8. **h** MPI (a.u.) in MCA area, FITC dextran. LPS 26% lower on average. Unpaired t-test, 95% CI of difference = − 43 to − 8.9. **i** MPI (a.u.) in area corresponding to olfactory bulb, BSA Alexa 647. Unpaired *t* test, 95% CI of difference = − 57 to 16. **j** MPI (a.u.) in area corresponding to olfactory bulb, FITC dextran. Unpaired *t* test, 95% CI of difference = − 37 to 14. Mean pixel intensity in arbitrary units, MPI (a.u.). Data are shown as mean and plotted individual samples, with *P* values above comparative brackets. Scale bars 6 mm

Stereoscopic examination revealed tracer distribution in cerebellum, general cortex, and olfactory bulb (measurement areas in Fig. 1a). Cortical distribution corresponded to area around the MCA. In LPS-treated mice, cortical tracer signal, measured as mean pixel intensity (MPI), was lower (although not quite significantly so in BSA-Alexa 647), compared to control animals (unpaired *t* test, 95% CI of difference = − 48 to 0.57 and − 35 to − 2.6, respectively; Fig. 1e, f). When we only measured an area corresponding to the perivascular space around the MCA, thereby omitting proximity to the injection site, tracer signal differed even more between treatment groups (31% and 26% lower in the two tracers, unpaired *t* test, 95% CI of difference = − 57 to − 5.8 and − 43 to − 8.9, respectively; Fig. 1g, h). Tracer intensity in olfactory bulb and cerebellum were not significantly different between groups (Fig. 1c, d, i, j).

**Fig. 2:**
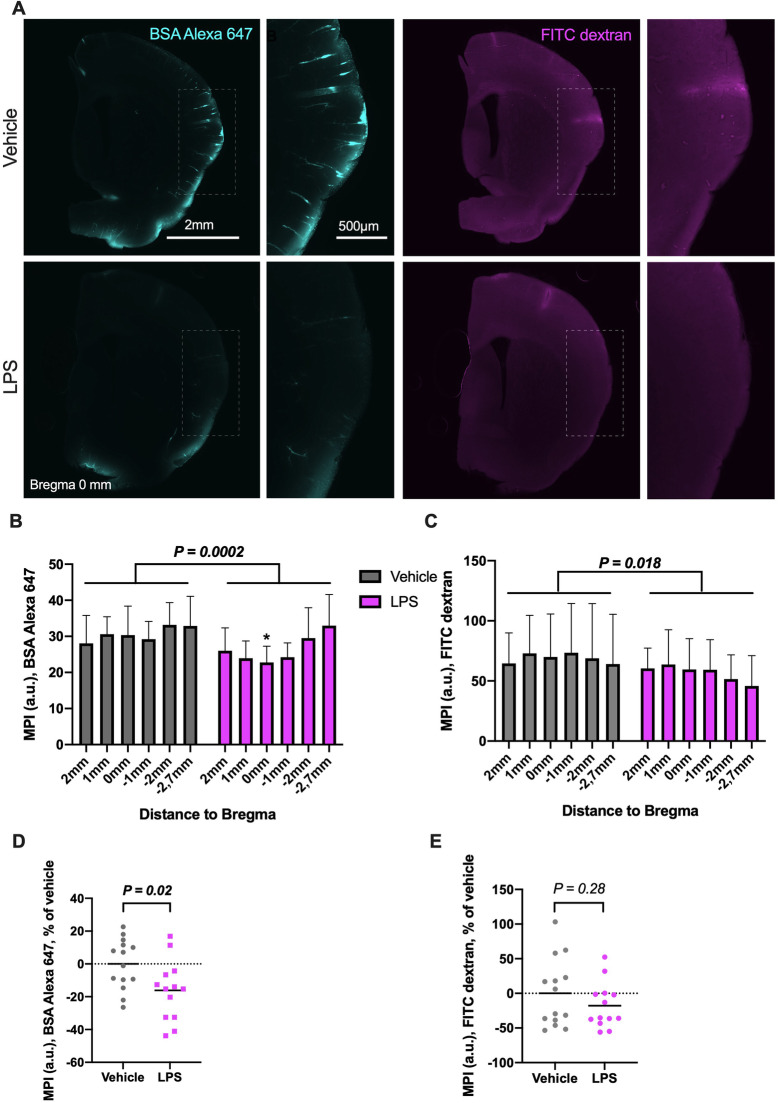
CSF-tracer penetration in coronal slices 3 h after systemic LPS-injection (*n* = 14), compared with vehicle controls (*n* = 13). **a** Tracer intensity in coronal slices, at bregma 0 mm. FITC dextran, being a much smaller and not as easily fixed molecule compared to the previous tracer, appears blurrier in slice images. **b** Two-way ANOVA, BSA Alexa 647 mean pixel intensity in arbitrary units, MPI (a.u.), treatment difference *P* = 0.0002. At bregma 0 mm, tracer signal was significantly lower in LPS-mice, multiple comparison with Šidák correction. **c** Two-way ANOVA, area of FITC dextran MPI (a.u.), treatment difference *P* = 0.018. **d** Average BSA Alexa 647 tracer MPI (a.u.) per animal, normalized to vehicle mean. LPS 16% lower on average. Unpaired *t* test, 95% CI of difference = − 30 to − 2.8. **e** Average FITC dextran tracer MPI (a.u.) per animal, normalized to vehicle mean. LPS 18% lower on average, albeit not significantly so. Unpaired *t* test, 95% of difference = − 51 to 16. Mean pixel intensity in arbitrary units, MPI (a.u.). *X*-axis indicates distance to bregma. Means are shown as staples with error bars = SD (**c**, **d**), as well as mean with plotted values (**e, f**). Scale bars 2 mm and 500 μm. *P* values above comparative brackets. * indicate multiple comparison difference *P* < 0.05

We then collected six 200-μm-thick coronal sections of the brains at bregma 2, 1, 0, − 1, − 2, − 2.7 mm, which exhibited tracer influx along cortical arterioles. LPS-treated mice showed a lower BSA Alexa 647 and FITC dextran tracer distribution in the brain, compared to control animals (two-way ANOVA, *P* = 0.0002 and *P* = 0.018, respectively; Fig. 2a–c). At bregma, the decrease was most pronounced with 25% lower BSA Alexa 647 tracer penetration in the LPS-treated mice (95% CI = − 48 to − 2.2; Fig. 2b). On average, tracer penetration was 16% (BSA Alexa 647) and 18% (FITC dextran) lower in LPS animals (albeit not significant for the FITC-measurement; unpaired *t* tests, 95% CI of difference = − 30 to − 2.8, and − 51 to 16 respectively; Fig. 2d, e).

Taken together, observations from CSF tracer experiments suggests that LPS decreases perivascular flow of CSF, as well as penetration of CSF into the brain parenchyma.

### Surgical monitoring

#### LPS-exposure changes heart rate, but not cerebral blood flow, respiration, or core temperature

**Fig. 3:**
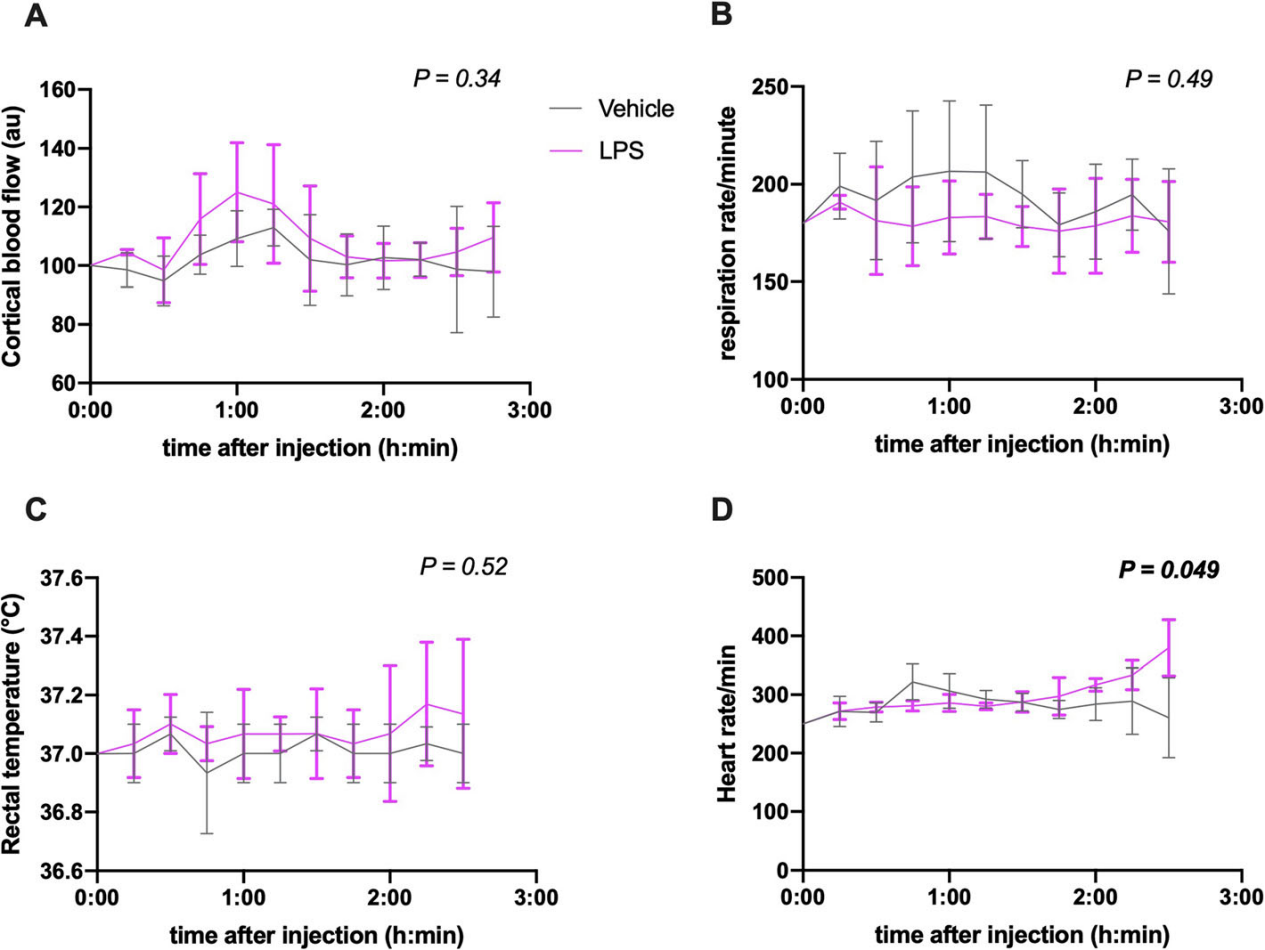
Cortical blood flow, respiration rate and rectal temperature within 3 h of systemic LPS injection. Vehicle *n* = 3, LPS *n* = 3. **a** Cortical blood flow in arbitrary units (au), measured with laser Doppler flowmetry. Two-way ANOVA, treatment factor 95% CI of difference = − 9.4 to 22. **b** Respiration rate measured with surgical monitor. Two-way ANOVA, treatment factor 95% CI of difference = − 52 to 30. **c** Basal temperature measured rectally. Two-way ANOVA, treatment factor 95% CI of difference = − 0.30 to 0.18. **d** Heart rate measured with surgical monitor ECG. Mixed-effects analysis (Two-way ANOVA), treatment factor 95% CI of difference = 0.09 to 28. Data are shown as mean and SD (error bars). *P* values for treatment factor differences

Because physiological parameters such as cerebral blood flow, heart rate, and respiration have been linked to CSF movement in the brain [16–20], we wanted to measure these variables using laser Doppler flowmetry and surgical monitoring after LPS-injections. Cortical blood flow measurements with laser Doppler showed no significant differences after acute LPS treatment, tested with two-way ANOVA (Fig. 3a). Simultaneously, we recorded respiration, temperature, and heart rhythm using a surgical monitor. During the several-hour long anesthesia, curves from LPS and control mice were stable and changed similarly, most likely because of additional ketamine injections (Fig. 3b, c). Heart rate, however, was significantly increased within 3 h of systemic LPS-administration (two-way ANOVA, 95% CI of difference = 0.09 to 28; Fig. 3d).

These results indicate that within 3 h of systemic exposure, LPS does not affect cerebral blood flow or respiration, but might affect heart rate.

### Immunohistochemistry, Western blot, and ELISA

#### LPS does not affect astrocytic AQP4 and GFAP within 3 h

**Fig. 4:**
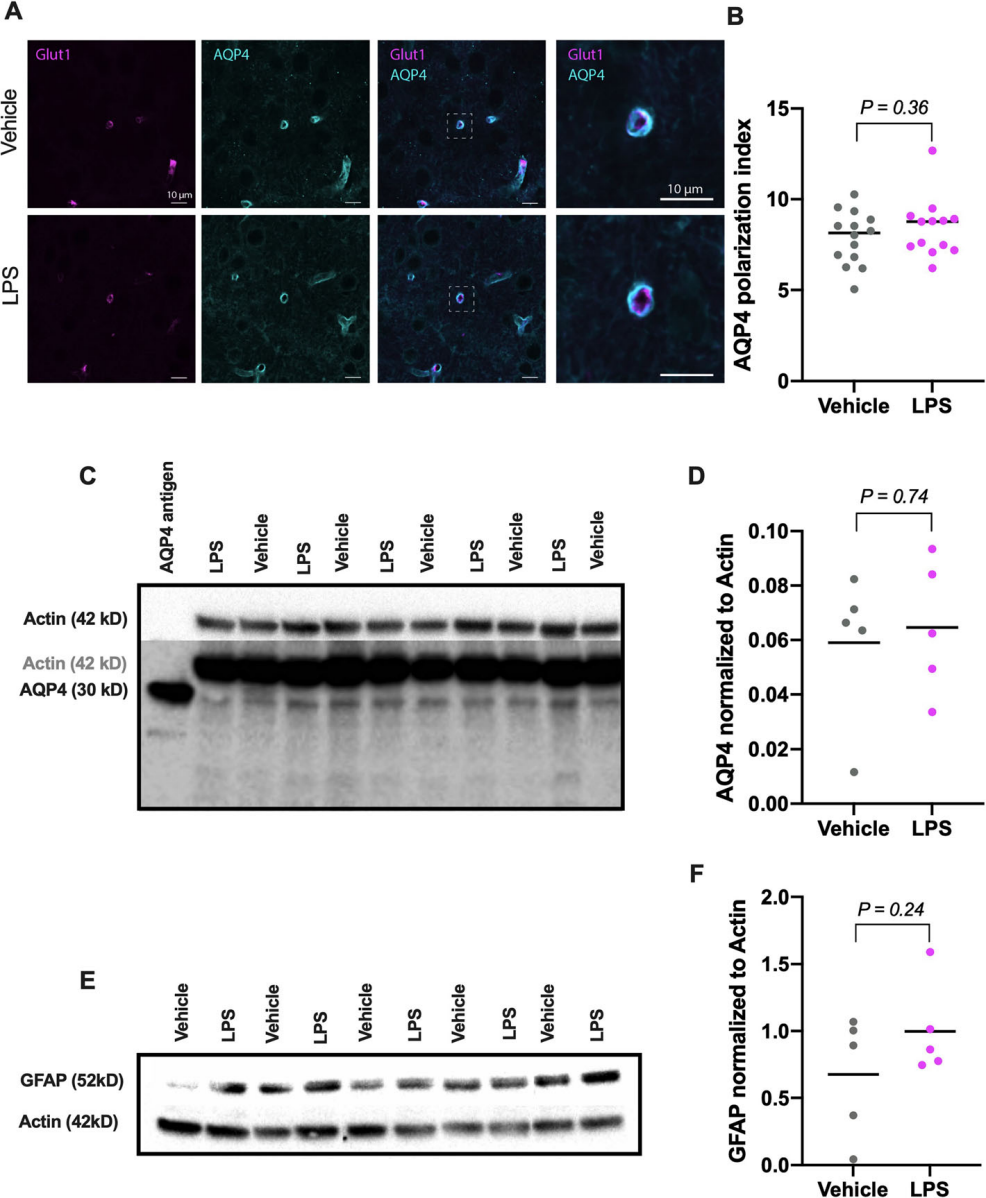
AQP4 expression (vehicle *n* = 5, LPS *n* = 5) and polarization (vehicle *n* = 14, LPS *n* = 13), as well as GFAP expression (Ctrl *n* = 5, LPS *n* = 5) after 3 h of systemic LPS injections. **a** AQP4 and Glut1 show cerebral vessels. **b** AQP4 polarization index differences were not observed 3 h after LPS. Unpaired *t* test, 95% CI of difference = − 0.67 to 1.8. **c** AQP4 detected bands at 30 kD. **d** Changes in AQP4, measured with Western blot, were not observed 3 h after LPS. Unpaired *t* test, 95% CI of difference = − 0.032 to 0.044. **e** GFAP showed bands at 52 kD. **f** GFAP was not significantly upregulated in LPS-treated mice. Unpaired *t* test, 95% CI of difference = − 0.26 to 0.91. Scale bars 10 μm

Since glymphatic function is tightly linked to AQP4 expression and astrocytes, we wanted to measure if LPS changed AQP4 expression in astroglial endfeet and expression of general astrocyte marker GFAP. Immunofluorescence in cortical and hippocampal areas revealed AQP4 to be expressed in astrocytic endfeet localized around blood vessels (Fig. 4a). AQP4 polarization, defined as vessel intensity to parenchyma ratio, did not significantly differ between LPS and control animals (unpaired *t* test, 95% CI of difference = − 0.67 to 1.8; Fig. 4b).

Immunoblotting of AQP4 and GFAP in cortical and hippocampal homogenates showed the presence of a band at 38 kDa and GFAP at 52 kDa, respectively, with no detectable difference between LPS and control mice (unpaired *t* test, 95% CI of difference = − 0.032 to 0.044, Fig. 4c, d; − 0.26 to 0.91, Fig. 4e, f).

#### LPS changes microglia morphology, but not microglial numbers or expression of galectin-3 after 3 h

**Fig. 5:**
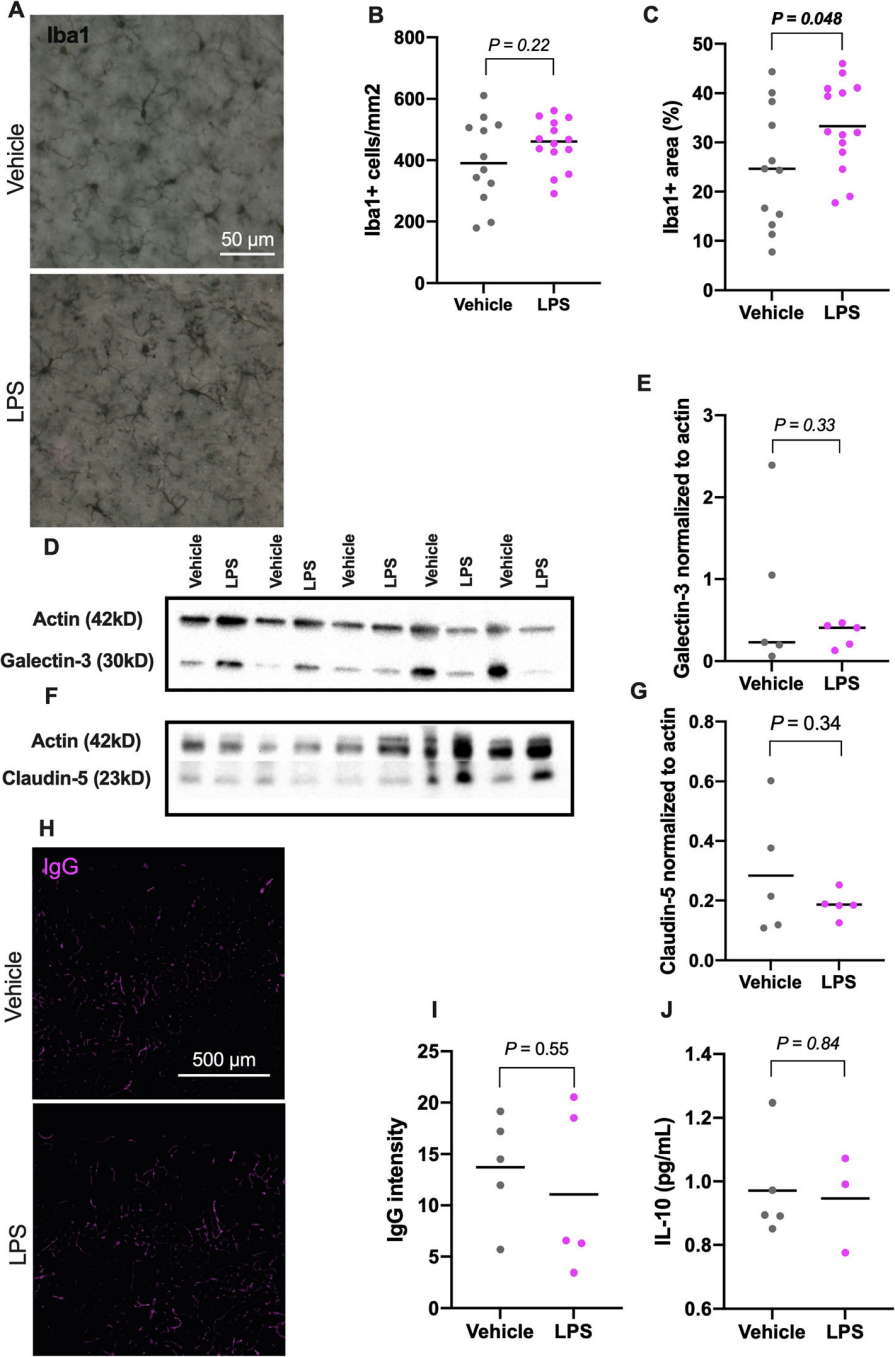
Markers of microglia, BBB permeability and cytokines. **a** Iba1^+^ cells in coronal cortical sections. Scale bar = 50 μm. **b** Statistical analysis of Iba1^+^ cell numbers 3 h after LPS injection. Vehicle group *n* = 13, LPS *n* = 14. Unpaired *t* test, 95% CI of difference = − 36 to 150. **c** Statistical analysis of Iba^+^ area in LPS injected animals. Unpaired *t* test, 95% CI of difference = 0.078 to 17. **d** Galectin-3 detects bands at 30 kD in both groups (vehicle *n* = 5, LPS *n* = 5). **e** Statistical analysis of galectin-3 WB, 3 h after systemic LPS exposure. Unpaired *t* test, 95% CI of difference = − 1.5 to 0.56. **f** Claudin-5 label bands at 23 kD. Vehicle *n* = 5, LPS *n* = 5. **g** Statistical analysis of Claudin-5 WB. Unpaired *t* test, 95% CI of difference = − 0.32 to 0.12. **h** IgG labeling reveal cortical vessels. Scale bar = 500 μm. **i** Statistical analysis of IgG intensity. Vehicle group *n* = 5, LPS *n* = 5. Unpaired *t* test, 95% CI of difference = − 12 to 7.1. **j** IL-10 mesoscale finds eight samples within detection range (vehicle *n* = 5, LPS *n* = 3). Treatment differences with unpaired *t* test, 95% CI = − 0.31 to 0.26. Data are shown as mean and plotted individual samples, *P* values for group comparisons are shown above brackets

In cortical and hippocampal areas, Iba1 antibody labeled microglial cells and their ramified processes. No significant differences were found in cell numbers between LPS and control mice quantified from cortex (unpaired *t* test, 95% CI of difference = − 36 to 150; Fig. 5a, b). However, the Iba1^+^ area was larger in the LPS mice compared to controls (unpaired *t* test, 95% CI of difference = 0.078 to 17; Fig. 5c).

Immunoblotting of galectin-3 (a marker for activated microglia) in cortical and hippocampal homogenates showed the presence of a band at 30 kDa with no difference between LPS and vehicle-treated mice (unpaired *t* test, 95% CI of difference = − 1.5 to 0.56, Fig. 5d, e).

#### LPS does not change BBB permeability markers and cytokines within 3 h

Measuring the tight junction protein claudin-5 with immunoblotting in cortical and hippocampal homogenates did not reveal any significant differences between treatment groups (unpaired *t* test, 95% CI of difference = − 0.32 to 0.12; Fig. 5f, g). Analysis of blood brain barrier permeability with IgG in cortical areas showed labeling in cerebral vessels in both vehicle and LPS injected animals with no statistical differences in intensity (unpaired *t* test, 95% CI of difference = − 12 to 7.1, Fig. 5h, i).

We measured neuroinflammatory cytokine expression 3 h after systemic LPS injection. Almost no signals were given for the 10 proinflammatory analytes (IFN-γ, IL-1β, IL-2, IL-4, IL-5, IL-6, IL-8, IL-10, IL-12, and TNF-α). Eight samples were above detection threshold, and only for IL-10. These eight samples, five vehicle controls and three LPS-treated brain homogenates, showed no group differences (unpaired *t* test, 95% CI of difference = − 0.31 to 0.26, Fig. 5j).

Altogether, our experiments of acute systemic LPS exposure did not generate detectable changes with regards to astrocytes, BBB permeability markers, and neuroinflammatory cytokines. We did, however, observe an increase in Iba1^+^ area, indicating microglial reactivity [21].

## Discussion

This study shows significantly decreased perivascular CSF tracer flow as early as 3 h after systemic exposure to LPS in male mice. The reduction was also visible in analysis of CSF tracer penetration into brain parenchyma. This rapid response upon LPS challenge on perivascular flow seems to occur earlier than we can detect an effect on many inflammatory readouts such as astrocytic response and a rise in cytokine levels.

These results may be relevant for several reasons. A decreased flow of CSF has been implicated in many diseases of the brain, such as Alzheimer’s disease, traumatic brain injury, and cerebral small vessel disease [4, 22–24]. Non-cleared aggregates are often highlighted in this correlation, but impaired CSF flow and neurodegeneration might also be linked by inflammation. In fact, a link between the CSF and the peripheral immune system has previously been described by Louveau et al. (2018), showing that the drainage of CSF and immune cells into cervical lymph nodes through lymphatic vessels is key for the development of experimental autoimmune encephalomyelitis (EAE), a neuroinflammatory condition and animal model of multiple sclerosis [25].

Systemic exposure to the bacterial endotoxin, LPS, has been shown to elicit cognitive dysfunction in rodents [26] — which is believed to have neuroinflammatory causes [27]. In fact, our results could thus indicate another possible mechanism to LPS-induced cognitive impairment—namely a decrease in CSF movement. This assumption, however, essentially rests on observed effects in later time points of LPS-exposure, as well as behavioral data, neither of which we have.

As briefly mentioned in the introduction, Erickson et al. observed that repeated systemic injections of LPS (3 mg/kg) lowered intracerebroventricularly injected CSF tracers in serum [4]. In our study, mice only received a single LPS injection, and at a lower concentration (1 mg/kg). While Erickson studied more parameters with very interesting results, including decreased amyloid beta efflux after LPS exposure [4], our study used a less invasive method of CSF tracing [9]. Since intracerebroventricular injections may cause inflammation, they could influence CSF flow readouts [28]. Observations even suggest that inserting a cannula through the cortex decreases glymphatic flow [29]. In contrast, tracers injected into the cisterna magna does not penetrate any brain tissue and thus does not cause parenchymal gliosis [9]. Furthermore, as opposed to sampling CSF efflux in serum [4], we used tracer imaging and show the decreased flow of CSF in the brain. However, our results are in line with the previous findings of Erickson et al. [4], which we find assuring.

Interestingly, a 2017 study by Karimy et al. showed that an intraventricular hemorrhage-induced and TLR-4 dependent inflammation caused CSF hypersecretion from the choroid plexus epithelium [30]. Since TLR4 is the main receptor for an LPS-response in mice [31], it could be possible that our effect was caused by an TLR4-mediated *oversecretion* of CSF, impairing glymphatic flow. However, such a mechanistic explanation warrants further studies, both with regards to how CSF oversecretion impacts glymphatic function, and how systemic LPS affects TLR4 in the choroid plexus.

In our mice, we tried to elucidate other possible mechanisms behind our observed effect on CSF movement. AQP4-expression and polarization in astrocytic endfeet have been shown to be key factors for CSF tracer dynamics [29], as well as to be affected after a neuroinflammatory stimuli [32], such as LPS [33]. Thus, we measured both AQP4 expression and polarization, but could not find any indication that our acute effects of LPS were caused by changes to this water channel. We have previously shown that galectin-3 can contribute to full-blown inflammatory microglial response 6 h following LPS challenge in vitro [34], and be a detrimental component in Alzheimer’s disease pathogenesis [35]. For this cohort with readouts 3 h post LPS exposure, we did not detect any differences in microglial activation marker galectin-3. Nor did we find any differences in astrocytic GFAP, BBB permeability markers, or an elevation of proinflammatory cytokines. Further studies are needed to evaluate the differences in these glial and cytokine responses at a later time point. We chose to set measurements at 3 h post LPS injections, since according to most published observations, that is approximately when the brain starts to be measurably affected — with neuroinflammatory markers such as IL-6, IL-1b, and TNF-alpha showing elevation [3]. However, not all reports show significant differences in markers of inflammation at our time point, not even at higher LPS doses. The LPS treatment was likely not without neuroinflammatory effects, since our LPS-treated mice displayed typical sickness behavior, which is closely tied to brain cytokine levels [36, 37]. Moreover, LPS-injected mice did show an increase in Iba1^+^ area, which indicates microglial reactivity to inflammatory stimuli [21].

Little is known about the interaction of microglial activation and CSF dynamics. Our recorded correlation might suggest either a role of microglial reactivity in abnormal CSF flow, or of the latter as a stimulus for microglial activation. Naturally, this interrelationship may not be more than two separate downstream effects of LPS exposure.

Glymphatic CSF flow has been shown to correlate with the cardiac cycle [16], and to follow the pulse-rhythms of cerebral arteries [16]. We therefore investigated the effect of LPS on cerebral blood flow, but did not detect any changes, in contrast to a previous report with intravenous administration of LPS [17]. When measuring cerebral blood flow, we simultaneously recorded respiration and heart rate, both of which are believed to affect CSF flow [18, 19]. Of these two parameters, we observed a statistically significant elevation in heart rate after LPS-injections, similar to what was measured by Ehrentraut et al. [20]. Since heart rate is believed to be one of the factors that makes glymphatic influx sleep dependent (heart rate low, and influx high, during deep sleep [19]), this LPS-induced effect on heart rate could then, at least partly, explain our detected LPS-effect on CSF influx. However, these results must be interpreted with caution due to the low number of subjects.

The strengths of this study are the steady differences in CSF-tracer intensity, using two tracers and analyzing with an established method as described in the literature [9, 10]. Limitations, other than what has been mentioned previously, are the use of only young male mice, since both age and sex are known factors for LPS response in mice [3, 38]— which may then have influenced our findings. Analyses of plasma markers may have aided us in finding interesting correlations. The same is true for blood pressure measurements, since arterial blood pressure is known to affect CSF flow [16, 39], and to be affected by LPS exposure [40]. Moreover, some experiments in our study, such as the physiological readouts (primarily because animals died under the long anesthesia), but also our Western blots and IgG measurements, were underpowered, increasing the risk of getting both false positive, and negative, results.

## Conclusion

In conclusion, our study reports another physiological response after acute exposure to the bacterial endotoxin, LPS, namely the significant decrease in perivascular distribution of CSF. We welcome future studies that endeavors to replicate, and thus confirm these findings—as we believe that they may help us grasp how inflammation affects the brain, in both health and disease.

## Data Availability

Raw data can be accessed upon request by contacting the corresponding author.

## Abbreviations

LPS: Lipopolysaccharides
KX: Ketamine and Xylazine
AQP4: Aquaporin-4
CSF: Cerebrospinal fluid
GFAP: Glial fibrillary acidic protein
Iba1: Ionized calcium-binding adapter molecule 1
PBS: Phosphate-buffered saline
T20: Tween-20
TX: Triton X100
MPI (a.u.): Mean pixel intensity in arbitrary units
